# Unemployment Insurance

**Published:** 1912-11-09

**Authors:** 


### NATIONAL INSURANCE ACT DEPARTMENT.

### XXXIV.—Unemployment Insurance—(*continued*).

We showed in our last article that an endeavour had been made to reduce the rate of unemployment by inserting in the Act a provision whereby an employer is allowed a refund of one-third of his own contributions paid to the unemployment fund on his own account in respect of a workman who has been continuously in his service for a year, and for whom at least forty-five contributions have been paid during that period.

A still more potent provision inserted in the Act with the same object in view now claims attention. The provision is contained in Section 96, and its character is clearly indicated by the marginal reference—namely, "Refund of contributions paid in respect of workmen working short time." In order to obtain the concession allowed the employer has to show to the satisfaction of the Board of Trade that during a period of depression in his trade he has systematically employed workmen on short time, and that he has paid the full contribution without making the deduction of the workmen's proportion from the wages paid. The form of the concession, if allowed, will be the refund of the whole of the contributions paid by the employer, on his own account and on behalf of the workmen, during the period of short work. No precise regulations are contained in the Act as to the conditions that must prevail before the concession can be allowed, except the provision that it will not be allowed when the hours of work have exceeded five-sixths of the number usually recognised as constituting a full week's work at that time in the trade and district. This provision is itself qualified by an exception which would seem to allow the refund when the method of effecting the short time work has been by stopping work on some day of the week usually recognised as a working day of at least four hours in the trade and district.

It is easy to see that without more precise regulations there might be difference of opinion between the employer and the Board of Trade as to the appropriateness of the circumstances to the application of the concession, and it is therefore laid down that an employer who seeks the advantage of the section may apply to the Board of Trade in advance, stating the facts of the case and requesting their ruling. If the ruling is in favour of its application the employer can, doubtless, rely on the refund being allowed, provided he adheres to the plan of carrying out the reduction of working hours approved by the Board of Trade, although there is nothing in the Act itself to make their decision final.

We can now see that the object of the provision is to induce the employer in periods of depression to keep his workmen engaged on short time, rather than to discharge some of his hands and maintain others on full time. The plan contemplated has the advantage of relieving the unemployment fund of claims for benefit which would presumably arise if men are discharged, and thus there are justifiable economic grounds for allowing the refund. Further than this, although the maintenance of workmen on part time may prove more expensive to the employer than the retention of a portion of his staff—and that, of course, the most efficient—on full time, the refund, which, in the normal case, is equivalent to 5d. a week for each workman, is a consideration which may well turn the scale in favour of the scheme. The advantage from the point of view of the workman, who would otherwise be thrown out of employment, is the difference between the reduced part-time wage and the unemployment benefit, which difference, it may be assumed, would be greatly in his favour.

The only person who may suffer is the efficient workman, who, in the worst periods of depression, could reckon on his services being in constant request for full-time labour. Seeing, however, that even the most efficient workman can only maintain his full vigour for a limited number of years, and that the time may come when, in similar circumstances, he would be discharged while a younger man would be retained, it would appear that even he has no real ground for complaint.

The provision has, therefore, much to commend it, and its application will be watched with interest.

A further inducement towards reducing the rate of unemployment is contained in the provision by which a workman, or his personal representatives in the event of his death, can claim in certain circumstances the return of the excess of his own contributions over the amount he has received in unemployment benefit, with compound interest at the rate of 2½ per cent. per annum, calculated in the prescribed manner. The conditions precedent to this repayment are that at least 500 contributions must have been paid—that is to say, contributions must have been paid for at least ten years, and the workman must have attained the age of sixty. This allowance partakes of the nature of a bonus to those workmen who for a long period of years are not a charge on the unemployment fund, and provides a means of saving small sums weekly to be drawn in old age. It must, however, be pointed out that the allowance had to be taken into account as one of the benefits in framing the actuarial estimates of the contribution required. The charge is, however, comparatively slight, and if no such repayment had been able to be claimed the rate of contribution could not have been reduced below the rate required under the Act as it at present stands. The inclusion of the benefit absorbed a considerable margin that could usefully have been preserved as a provision against the ordinary benefits, and the only ground on which it can be justified is that the semblance of equity to those who contribute but do not claim the unemployment benefit goes a long way towards reconciling such workmen as have no prospect of requiring the unemployment benefit to the new legislation.

One further point has to be taken into account in connection with this provision—namely, that, although the repayment may have been allowed at the age of sixty, this does not relieve the employer from contributing on behalf of such workman who still remains in his employment after having received such repayment. The question then arises as to the right of the workman to claim unemployment benefit after he has received the return of his excess of contributions. The rate of benefit is not affected, but the period for which it may be drawn will be reduced, so that instead of the whole of the contributions paid before the repayment was received, only five-eighths of such contributions will be taken into account.

We are now in a position to show the relation between the contributions and benefits according to the actuarial estimates.

The average annual contribution is made up as follows:—

| | Per annum £ | s. | d. |
|---|---|---|---|
| **From workman (44 weeks at 2½*d.*) ... ... ...** | **0** | **9** | **2** |
| **From employer, after allowing for refund for workmen continuously employed ... ...** | **0** | **7** | **10** |
| **From State... ... ... ... ... ... ...** | **0** | **6** | **1⅓** |
| | **1** | **3** | **1⅓** |
| **Expenses of administration (10 per cent.) ... ...** | **0** | **2** | **3⅝** |
| **Average contribution available for benefits ...** | **1** | **0** | **9⅝** |

The estimated average cost of the unemployment benefit, on the basis of 26.8 days unemployment per workman per annum throughout the insured trades, after making allowance for the conditions under which the payment of benefit is restricted and for the return of contributions at the age of sixty is about £1. There is, therefore, an estimated margin of about 10d. per member per annum, which is equivalent to about 4.3 per cent. on the net cost of the benefits. This is a very slight margin in view of the imperfect data upon which the estimates were framed, but it is hoped that the reserve that will accrue to the fund by reason of the waiting period of six months, during which contributions are payable but no benefits receivable, will enable the fund to continue financially sound for a long period.

### The State Subvention.

The expected cost to the State of the contributions to the unemployment fund is about £660,000 per annum, which will, of course, have to be borne by general taxation. Power is, however, reserved to the Board of Trade to extend the scope of the unemployment insurance to other trades so long as that extension is not calculated to require an addition to the State subsidy, which would bring the total amount to more than £1,000,000 a year for three years from the date of such extension. This limitation will probably have the effect of precluding the extension of the insured trades until such time as it has been possible to fully test the financial soundness of the scheme as at present in force. In addition to the direct contribution of the State to the unemployment fund provision is made whereby the State virtually guarantees the payment of the benefits, for if the fund should be insufficient to discharge its liabilities the Treasury is empowered to make advances out of the Consolidated Fund to the extent of but not beyond £3,000,000. This does not mean that the State guarantees the payment of the benefits at the present rate indefinitely, but that should occasion demand, through widespread unemployment, that advances should be made temporarily to enable the payments to be made, the State will come to the aid of the unemployment fund. If, whilst any part of such advance is outstanding, it appears to the Treasury that the unemployment fund is insolvent, the Treasury may direct a modification of the rates of benefit or an alteration of the rates of contribution. The rate of benefit must not, however, be reduced below 5s. a week, nor the contribution increased by more than 1d. a week each from the workman and the employer. In any case, such modification is to be of a temporary character, and is not to remain in force more than three months beyond the time that the advance from the State is repaid. Permanent changes in the rates of benefit or contribution may be made as the result of experience, but not more frequently than once in seven years.

The unemployment provisions of the Act are being administered through the Labour Exchanges, assisted in certain cases by the Trade Unions, and thus a very useful means of co-ordinating the agencies for the reduction of unemployment has been brought into operation.

An endeavour has been made to restrict litigation as far as possible by giving insurance officers powers to settle matters in dispute, with appeal to Courts of Referees constituted by equal numbers of employers and workmen with a chairman appointed by the Board of Trade. An umpire has also been appointed whose decision in matters relating to the fulfilment of the statutory conditions entitling to benefit is final.

